# Understanding the Ethical Issues of Brain-Computer Interfaces (BCIs): A Blessing or the Beginning of a Dystopian Future?

**DOI:** 10.7759/cureus.58243

**Published:** 2024-04-14

**Authors:** Efstratios Livanis, Polychronis Voultsos, Konstantinos Vadikolias, Panagiotis Pantazakos, Alexandra Tsaroucha

**Affiliations:** 1 Department of Accounting and Finance, University of Macedonia, Thessaloniki, GRC; 2 Postgraduate Program on Bioethics, School of Medicine, Democritus University of Thrace, Alexandroupoli, GRC; 3 Laboratory of Forensic Medicine & Toxicology (Medical Law and Ethics) School of Medicine, Faculty of Health Sciences, Aristotle University of Thessaloniki, Thessaloniki, GRC; 4 Department of Neurology, University Hospital of Alexandroupolis, Alexandroupoli, GRC; 5 Department of Philosophy, School of Philosophy, National and Kapodistrian University of Athens, Athens, GRC

**Keywords:** brain-computer interface (bci), brain-machine interface, review, neuroethics, ethics of bci

## Abstract

In recent years, scientific discoveries in the field of neuroscience combined with developments in the field of artificial intelligence have led to the development of a range of neurotechnologies. Advances in neuroimaging systems, neurostimulators, and brain-computer interfaces (BCIs) are leading to new ways of enhancing, controlling, and "reading" the brain. In addition, although BCIs were developed and used primarily in the medical field, they are now increasingly applied in other fields (entertainment, marketing, education, defense industry). We conducted a literature review following the Preferred Reporting Items for Systematic Reviews and Meta-Analyses (PRISMA) guidelines to provide background information about ethical issues related to the use of BCIs. Among the ethical issues that emerged from the thematic data analysis of the reviewed studies included questions revolving around human dignity, personhood and autonomy, user safety, stigma and discrimination, privacy and security, responsibility, research ethics, and social justice (including access to this technology). This paper attempts to address the various aspects of these concerns. A variety of distinct ethical issues were identified, which, for the most part, were in line with the findings of prior research. However, we identified two nuances, which are related to the empirical research on ethical issues related to BCIs and the impact of BCIs on international relationships. The paper also highlights the need for the cooperation of all stakeholders to ensure the ethical development and use of this technology and concludes with several recommendations. The principles of bioethics provide an initial guiding framework, which, however, should be revised in the current artificial intelligence landscape so as to be responsive to challenges posed by the development and use of BCIs.

## Introduction and background

The rapid development of neurotechnology brings opportunities and challenges to the medical and non-medical sectors. Of particular interest are the brain-computer interface (BCI) systems. BCIs are devices that create a direct connection between the brain and an external device. In its simplest version, BCIs consist of the user, arrays of multiple electrodes, a signal processor, and an application [1]. The development of BCIs was contributed in the 1920s by the German psychiatrist Hans Berger who was the first to record alpha and beta brain waves and is the inventor of electroencephalography (EEG) [2]. However, the term brain-computer interface system was first introduced in 1973 by Jacques Vidal, a professor of computer science at the University of California (UCLA) [3]. In the last decades, several research groups on BCIs have been established all over the world. In that regard, the field of application of BCIs has also been expanded for non-medical purposes. The evolution of even more advanced BCIs has been significantly enhanced by the development of machine-learning techniques that are used to process brain data. However, these interventions in the human brain raise questions about the benefits and risks, as well as issues of human dignity, personhood and autonomy, privacy and security, ethical and legal responsibility for decisions and actions based on BCIs, and accessibility to these technologies and social justice. In this paper, through a review of the relevant literature, we attempt to clarify the ethical issues of BCIs and highlight the actions that need to be taken for the ethical development and use of this technology in the medical and non-medical sectors.

## Review

Methodology

The literature search was conducted in December 2023 using the PubMed and Web of Science bibliographic databases. To identify the keywords used in our search, we looked at the keywords used in previous literature reviews [4,5]. Specifically, the following search queries were used (Table 1).

**Table 1:** Search queries.

Database	Search query
PubMed	(("brain computer interface*"[tiab] OR "brain machine interface*"[tiab]) AND (("humanity"[tiab] OR "personhood"[tiab]) OR ("user safety"[tiab] OR "safety"[tiab]) OR ("autonomy"[tiab] OR "personal autonomy"[tiab]) OR ("responsibility"[tiab] OR "liability"[tiab] OR "regulation"[tiab]) OR ("normality"[tiab] OR "stigma"[tiab] OR "social stigma"[tiab]) OR ("research ethics"[tiab] OR "consent"[tiab] OR "informed consent"[tiab]) OR ("privacy"[tiab] OR "security"[tiab] OR "brain hacking"[tiab]) OR ("justice"[tiab] OR "social justice"[tiab] OR "moral judgment"[tiab])))
Web of Science	(ab=("brain computer interface*" OR "brain machine interface*") OR ti=("brain computer interface*" OR "brain machine interface*")) AND (ab=(("humanity" OR "personhood") OR ("user safety" OR "safety") OR ("autonomy" OR "personal autonomy") OR ("responsibility" OR "liability" OR "regulation") OR ("normality" OR "stigma" OR "social stigma") OR ("research ethics" OR "consent" OR "informed consent") OR ("privacy" OR "security" OR "brain hacking") OR ("justice" OR "social justice" OR "moral judgment")) OR ti=(("humanity" OR "personhood") OR ("user safety" OR "safety") OR ("autonomy" OR "personal autonomy") OR ("responsibility" OR "liability" OR "regulation") OR ("normality" OR "stigma" OR "social stigma") OR ("research ethics" OR "consent" OR "informed consent") OR ("privacy" OR "security" OR "brain hacking") OR ("justice" OR "social justice" OR "moral judgment")))

**Figure 1:**
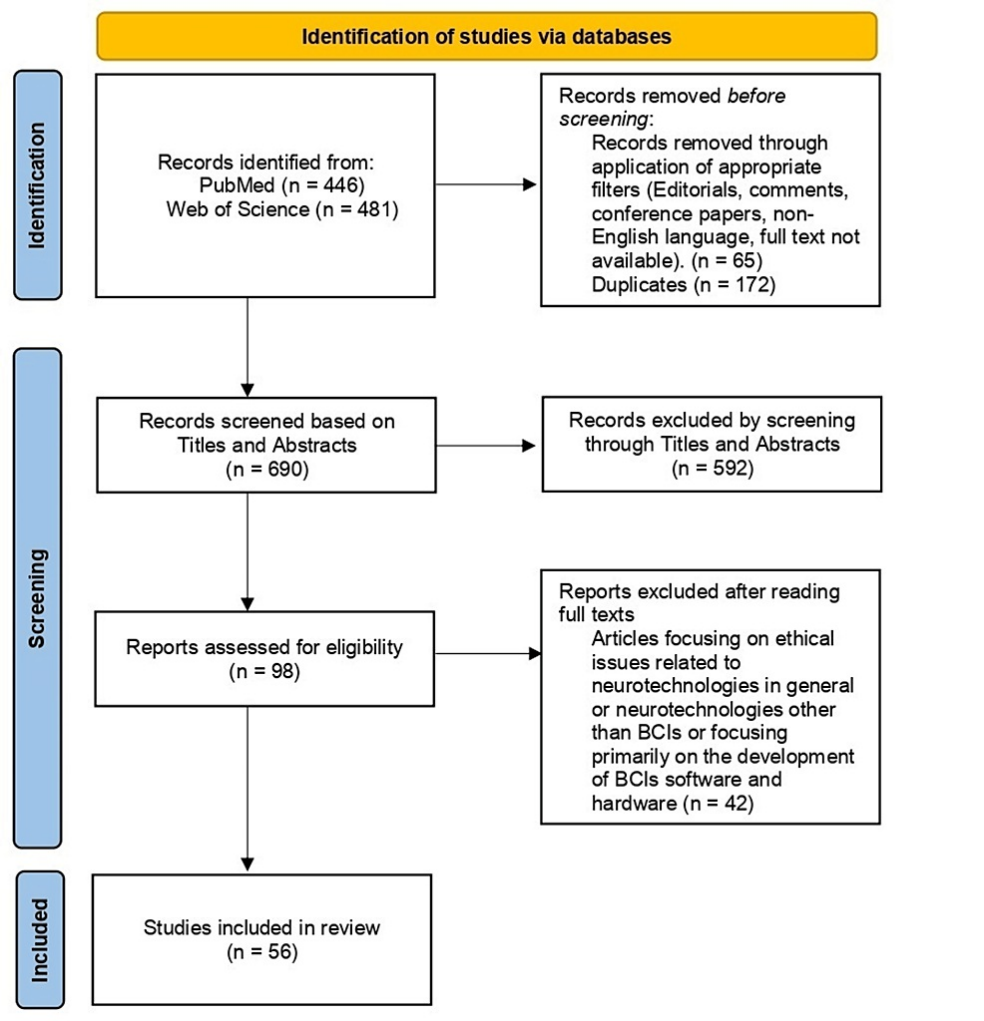
Systematic review PRISMA flowchart demonstrating the steps of searching, screening, and selecting data. PRISMA: Preferred Reporting Items for Systematic Reviews and Meta-Analyses

The following inclusion criteria were applied: peer-reviewed journal articles, articles with full manuscripts and English language, and articles addressing the ethical issues of BCIs in medical and non-medical fields. Editorials, comments, conference papers, articles in a non-English language, articles focusing on ethical issues related to neurotechnologies in general or neurotechnologies other than BCIs (e.g., deep brain stimulation, etc.), and articles that focus primarily on the development of BCI software and hardware were excluded. Preferred Reporting Items for Systematic Reviews and Meta-Analyses (PRISMA) guidelines [6] were followed throughout the entire process (Figure 1) to ensure quality and transparency. The search resulted in 446 studies on PubMed and 481 studies on Web of Science that met the query criteria. In the first step, 65 records were removed before screening, through the application of appropriate filters (editorials, comments, conference papers, non-English language, full text not available). Additionally, 172 duplicates were removed with EndNote. In the next step, titles and abstracts were screened by two independent reviewers (EL and PV). The full texts of the selected 98 articles were then examined by the reviewers. A consensus meeting with all the members of the research team was held to resolve any disagreements. This resulted in 56 articles being included in this review.

Quality Assessment

In the present systematic literature review, empirical studies (quantitative, qualitative, or mixed), reviews, and opinion/discussion papers were included. The Mixed Methods Appraisal tool [7] was used to assess the quality of quantitative, qualitative, or mixed empirical studies. Furthermore, as there is no established best practice approach for quality assessment in review/opinion/discussion papers of normative literature [8], review/opinion/discussion articles published in a peer-reviewed journal cited in PubMed or in a peer review journal published by a reputable academic publisher was taken as a satisfactory indicator of quality to justify inclusion in the review. Two independent researchers (SL and PV) assessed the quality of the included studies. Disagreements between the reviewers on the quality of the included studies were resolved through discussion.

Analysis of ethical issues

User Safety

The issue of user safety refers to the potential health risks to the user of both invasive and non-invasive BCIs. The health of the user may be negatively affected both immediately and in the long term by the use of the device [9]. Injuries to the user may result from the insertion of electrodes into the cerebral cortex, loss of functional electrodes over time, malfunctioning of the power supply to the implanted electrodes, and other intra-operative or post-operative complications [10]. Furthermore, according to the results of a survey of neurosurgeon groups in 32 countries around the world, it was found that participants appear highly skeptical and conflicted about safety issues [11]. However, non-invasive BCIs may have negative effects on neuroplasticity [12]. It may raise dilemmas about their use in younger age groups [13].

Humanity and Personhood

Many scholars seem to be concerned about the ethical dilemmas of humanity related to personality disorders due to the use of BCIs. Indeed, concerns about how personality might be affected in a transhumanist world arose as early as the 1920s, when Karel Chapek introduced the term 'robot' in a work presented in Prague in 1921 [14]. The integration of BCIs into the human body can complicate our concepts of social identity and our view of our bodies [15] and affect how others see us and how we perceive ourselves. A survey on public perceptions of BCIs in Germany [16] recorded, among other things, participants' concerns about personality changes.

To better manage personality issues, psychometric self-image measurements could be used before an experiment is initiated [17]. Moreover, the need for establishing a qualitative methodology to capture disturbances (in terms of identity and self-action) due to the application of invasive BCIs is suggested by other researchers [18,19].

There is also the concern that in the future developments in BCIs may replace the physical circuits in the brain. Thus, the brain can be turned into a completely artificial organ; that is, humans can be turned into 'cyborgs' [20]. On this occasion, a question arises as to how we should classify some people as humans or machines. The line of distinction between humans and machines is made blurry.

Autonomy

According to Burwell et al. [4], the issue of autonomy is primary as it is linked to other ethical dilemmas such as the determination of moral and legal responsibility. Although BCIs can restore autonomy or part of it to users with mobility or communication impairments, concerns remain as to whether the actions achieved through BCIs are the result of actual autonomous action. There is a view that BCI-mediated action has some characteristics that distinguish it from ordinary behavior, and for this reason, some revisions could be made to theories of action and to the definition of the concept of agency [21].

As Goering et al. [22] suggested, we could move from a view of unaffected and exclusively autonomous action to a social/relational view where we acknowledge the influence of people around us (such as patient caregivers, family, etc.) and devices on our behavior. Relying or depending on others to carry out an action does not necessarily mean a reduction in our agency. Michałowska et al. [23] argued that the issue of the potential limitation of a BCI user's autonomy could be addressed by adopting a broader concept of autonomy that does not require all decisions to be completely free. We could consider that action in the decision-making process based on the user's wishes and preferences would be sufficient to ensure personal autonomy.

Stigma and Normality

BCIs can help people with disabilities, such as mobility impairments, deafness, and communication disorders to overcome social exclusion. However, using non-invasive BCIs can sometimes stigmatize users [24]. Furthermore, the general adoption of these systems also for people without disabilities can reduce stigmatization, but it can widen the stigmatization of people with disabilities who will not use these systems, especially from the perspective of a transhumanist future [25,26]. The conspicuous use of these devices could make the user's medical problem more apparent or even the user can be treated by society as a 'cyborg' [27]. Additionally, the use of BCIs for medical purposes and, even more so, the use of BCIs for commercial purposes can influence social norms [28] of what is considered normal.

Privacy and Security

The use of BCIs raises important issues regarding the privacy and security of their users. In the scientific literature, there are several papers, with an increasing trend over the last years, showing that brain data could be extracted through malicious external action or there could be a case of unauthorized access to the functions of BCIs with further impact on the issues of autonomous action and liability [29-32]. Indeed, the issue of malicious external attacks on the brains of BCI users could be described as a kind of 'neurocrime' [33]. However, BCI systems security is not yet mature, creating opportunities for malicious external actions [34]. At the same time, the issue of biocybersecurity in the field of clinical neuroscience is highlighted through the case of vulnerabilities that both BCIs and hospital neuro-devices may have [35].

Concerns about privacy issues and the risk of brain data being used for financial gain by companies were also expressed by rehabilitation professionals in Spain in a related survey [36]. Furthermore, in the area of psychiatry, the possible future use of BCIs to read thoughts and feelings may raise issues of confidentiality and suspicion [37].

Research Ethics and Informed Consent

Scholars emphasize their concerns that the use of BCIs can violate research ethics and informed consent principles. In patients with locked-in syndrome, the morality of the use of BCIs requires more analysis and collaboration between the parties involved [38]. In the context of informed consent, it is important that all potential risks are analyzed in a way that is understandable to the user [39]. Although the responsibility for informed consent lies with the researcher, the responsibility for identifying and investigating risks belongs to all participants in the BCIs field [9].

Ethical concerns also arise regarding the timing of the completion of clinical trials. The timing of termination of BCI studies may not be clear as participants may not want to stop and researchers may want to continue to collect brain data and adjust algorithms accordingly [40]. Some researchers express concern about issues of transparency, bias, ethics of algorithms, and ethics of combination of BCIs with artificial intelligence [41-43].

The way BCIs are presented in the media can also pose problems for informed consent. It has been observed that the majority of the media emphasize the positive aspects of BCIs rather than the negative ones, thus creating unrealistic expectations and downplaying safety issues [44]. Other concerns are related to researchers’ responsibility towards participants in their studies [45].

Finally, it is worth mentioning the results of a study conducted by Sullivan et al. [46] who considered the 'ethical language' used by publications when referring to BCI research in humans. They identified ‘ethical language’ in 76% of the papers reviewed, with percentages being significantly lower in neuroengineering journals.

Responsibility and Regulation

Determining the degree of user liability for acts performed using BCIs is mentioned in academic literature, which highlights the need to update the institutional and regulatory framework, so as to address legal issues arising from the use of BCIs [47]. Bublitz et al. [48] argued that BCI users can be held liable in three situations: 1) for harmful actions performed during the use of BCIs, 2) for failing to prevent harmful effects that resulted from the use of BCIs, or 3) for foreseeable harmful outcomes of the implementation of BCIs. Another bet is to identify the party that is deemed liable in case of a BCI malfunction. Is the user, the BCI process itself, the manufacturer, or a third party responsible? Schönau [49] proposed, for this case, a framework in which the range of liability is outlined according to the user's level of control per type of BCIs.

Davidoff [50] wondered to what extent BCIs accurately reflect the brain state of their users when they only record a small percentage and, consequently, whether BCI users should be held accountable for actions taken through BCIs if those actions do not reflect their full current brain state. Furthermore, Rainey et al. [51] distinguished the case where BCIs are used for medical purposes, in particular by people with disabilities, from the case where it is used for entertainment. In this latter case, they suggest that the attribution of responsibility to the user should be done with less strict criteria.

Another issue is how the law should address brain data. Rainey et al. [52] noticed that the categorization of brain data in the General Data Protection Regulation (GDPR) is not clear when these data are derived from non-medical devices. For the effective handling of brain data, Ienca et al. [53] proposed a framework that includes four pillars: specific regulation of brain data, ethical guidelines accompanied by relevant legislation, promotion of responsible innovation, and establishment of neurorights [54] within the framework of human rights.

Justice

The increasing use of BCIs by both patients and the healthy population raises concerns about the degree of equitable access [55] to these technologies and the risk of creating inequalities [56]. There is a risk that the technology of BCIs may not be accessible to all, especially to people with disabilities, due to high prices [25] or insurance companies' reluctance to cover the cost of BCIs. This leads to the ethical dilemma of whether society has a moral responsibility to provide equal access to assistive technology, such as BCI devices, for people with disabilities. Furthermore, there may be raised issues related to the context of criminal justice in case of crimes committed via BCIs. To this effect, it is to be investigated if and under what conditions the user of BCIs used it to commit an illegal act [57].

Enhancing Human Capabilities

Researchers expressed concern about the enhancement of human capabilities beyond the normal [11,58,59] and how this may affect various sectors of society. Attention is also needed in cases of neuroenhancement in the military [60] where soldiers could be considered a particularly vulnerable group [61]. It is also stated that particular consideration should be given as to whether there is a need for neuroenhancement. In addition, a cost-benefit analysis should be carried out, from a medical and military point of view [62]. Moreover, Moulin [63] reviewed the institutional framework regarding the participation of humans in experiments to enhance human abilities with BCIs. The author highlights the issue that some countries, such as China, have a different institutional framework. Thus, there is a risk that research centers in countries with a stricter institutional framework may choose to carry out research in countries such as China, bypassing the relevant restrictions in their home country.

Empirical studies on BCIs

In this literature review, empirical studies (both quantitative, qualitative, and mixed) have been identified over the last five years. Burwell et al. [4] published a systematic review in 2017 where they highlighted the need for further empirical research on ethical issues related to BCIs. The authors state that 'we likely require further empirical investigation into public hopes or worries, and into the emerging domains of BCI application' [4]. Williams et al. conducted a mixed empirical, cross-sectional, two-stage, mixed-method (qualitative and quantitative) study with neurosurgeons regarding the acceptability of risky invasive BCIs [11]. The study was conducted 'in keeping with the precedence within the literature' [64,65]. The authors concluded that, while 'augmentative BCI applications remain more controversial than rehabilitative applications', many neurosurgeons remain 'open to augmentative BCI' [11]. In Germany, Schmid et al. conducted a representative online survey and concluded that the findings of their study revealed 'a positive view and high level of trust in BCIs on the one hand, but, on the other hand, a wide range of ethical and anthropological concerns' [16]. In Spain, Monasterio Astobiza et al. [36] conducted a mixed-method (qualitative and quantitative) empirical study. The conclusion that 'a majority of rehab professionals either strongly or somewhat accept the use of brain-computer interfaces (BCI) in rehabilitation therapy' emerged from the quantitative stage of the study [36]. However, 'strong societal (and other concerns), attitudes and perceptions, against BCI technology use in their daily practice' emerged from the qualitative stage of the study [36].

BCIs and international relationships

It is argued in studies that BCI-related research has a considerable impact on the specific relationship between China and the United States [60,63]. The United States has indicated high interest and invested a lot of money in BCIs and, more broadly, in brain-related research [60,63]. Weak adherence to the (more prohibitive) international law and native supply of research monkeys are among the factors facilitating BCI-related research in China [60,63]. China has an advantage through BCIs in the military sector [60,63]. The superiority of the United States over other nations in the military field appears to be threatened because of the speed and manner in which related research is conducted in other countries [60,63]. The French Secretariat-General for National Defence and Security (SGDSN) stated, 'The situation of neurosciences’ military applications [is comparable with] the situation that existed in the nuclear field during the sixties' [63]. Moreover, China can have an advantage in the commercial sector where the advantage may 'lead to privacy issues for very personal data - the activity of a human brain and interpretations of that activity that give insight into mental state and mood' [60]. Furthermore, researchers should bear in mind that 'in the specific case of China, we have much more limited access to information about research spending and outputs compared to the United States' [60]. At any rate, it should be highlighted that Moulin et al. are not optimistic about reaching an international agreement on a commonly accepted regulatory framework for BCIs in the near future [63]. It is to be noted that the literature previous to the last five years does not discuss the effect of BCIs on international relationships. In 2020, Coin et al. stated that 'questions of BCI policy have not yet become a frequent point of discussion in the relevant literature on BCI ethics, and we argue this should be addressed in future work' [5].

Recommendations

**Figure 2:**
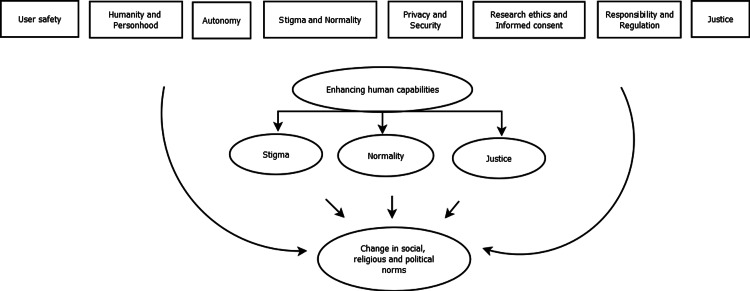
Categorization of ethical issues from the development and use of brain-computer interface systems emphasizing the issue of enhancing human capabilities. This figure is an original work of the authors.

The ethical challenges mentioned above have drawn the attention of scholars and professionals, emphasizing the need to establish a global ethical framework for the development and use of BCIs. Moreover, Binkley et al. suggested the establishment of a new committee or regulatory authority to evaluate BCIs with respect to humanitarian principles and based on both individual and societal benefit [66]. Rainey et al. [52] argued that brain data collected from BCIs are as sensitive as medical data and, therefore, a multidisciplinary dialogue on the privacy and security of brain data is needed. Indeed, the process for obtaining informed consent should be multidisciplinary, systematic and transparent, ongoing, and exploratory [67]. A framework for active participation of users in the management of their brain data is also suggested [68]. Furthermore, BCI responses are derived from machine-learning algorithms that use a small fraction of brain activity as datasets. However, it is important to determine a specific percentage (relatively high) of brain activity that must be recorded for users to be held accountable for their actions [50]. It is also important to consider the development of an appropriate framework for the future use of BCIs in the criminal justice system for the protection of human rights. Additionally, safeguards need to be put in place to ensure that economically disadvantaged patients have equal access to BCIs, possibly through government financial assistance policies. Additionally, the bioethical issues regarding the enhancement of human capabilities through BCIs are likely to become even more pressing in the future for both academia and society. It is thus recommended to pay special attention to this issue by examining (Figure 2) these ethical concerns in relation to human existence and stigma; the concept of what is considered normal; fair access to neuroenhancement technologies; and, by extension, the potential change in social, religious, and political norms. Finally, it is important to embed ethics in neurotechnology research and education. The inclusion of bioethics courses in the curricula of non-medical faculties and related programs at all levels of education can assist in this direction so that even children and young people can become familiar with the principles of bioethics.

## Conclusions

BCIs represent a neurotechnology that has created significant expectations in the field of medicine. Furthermore, a significant number of BCIs have been developed for healthy people over the recent years. In this paper, a variety of distinct ethical issues were identified, which, for the most part, were in line with the findings of prior research. However, we identified two nuances, which are related to the empirical research on ethical issues related to BCIs and the impact of BCIs on international relationships. Not surprisingly, developments raise concerns about how far this technology can go and what impact it will have on social, political, and religious norms. In that regard, discussions regarding the commercialization of brain data and the potential for manipulation of the human brain are likely to increase in the coming years. It is crucial to consider the values and needs of future users during the development of BCIs. This requires continuous interaction between the scientists involved in the development of BCIs and the people whose lives may be affected by them. At the same time, the associated risks should be identified and understood while are not always clear to business people, investors, users, and society. It is therefore imperative that the scientific, political, and business communities cooperate in order to indicate, through bioethics, the safeguards that will ensure the ethical design of the development, use, and management of BCIs. It is everyone's ethical duty to ensure that technological development benefits humanity.
